# Impact of El Nino Southern Oscillation and Climate Change on Infectious Diseases with Ophthalmic Manifestations †

**DOI:** 10.3390/tropicalmed10100297

**Published:** 2025-10-18

**Authors:** Crystal Huang, Caleb M. Yeh, Claire Ufongene, Tolulope Fashina, R. V. Paul Chan, Jessica G. Shantha, Steven Yeh, Jean-Claude Mwanza

**Affiliations:** 1 Truhlsen Eye Institute, University of Nebraska Medical Center, Omaha, NE 68105, USA; crystal6@sas.upenn.edu (C.H.); calebmyeh@gmail.com (C.M.Y.); tfashina@unmc.edu (T.F.); 2 Department of Ophthalmology and Visual Sciences, University of Illinois Chicago, Chicago, IL 60612, USA; cufong2@uic.edu (C.U.); rvpchan@uic.edu (R.V.P.C.); 3 F.I. Proctor Foundation, Department of Ophthalmology, University of California San Francisco, San Francisco, CA 94107, USA; jessica.shantha@ucsf.edu; 4 Global Center for Health Security, University of Nebraska Medical Center, Omaha, NE 68198, USA; 5 Emory Eye Center, Emory University School of Medicine, Atlanta, GA 30322, USA; 6 Child Health Research Institute, University of Nebraska Medical Cetner, Omaha, NE 69198, USA; 7 Department of Ophthalmology, University of North Carolina, Chapel Hill, NC 27514, USA; jean-claude_mwanza@med.unc.edu

**Keywords:** El Nino Southern Oscillation, dengue, chikungunya, malaria, emerging infectious diseases, climate change, ophthalmology

## Abstract

Climate change and the El Niño Southern Oscillation (ENSO) events have been increasingly linked to infectious disease outbreaks. While growing evidence has connected climate variability with systemic illnesses, the ocular implications remain underexplored. This study aimed to assess the relationships between ENSO-driven climate events and infectious diseases with ophthalmic consequences. A narrative review of 255 articles was conducted, focusing on infectious diseases influenced by ENSO and their associated ocular findings. 39 articles met criteria for full review, covering diseases such as dengue, zika, chikungunya, malaria, leishmaniasis, leptospirosis, and Rift Valley fever. Warmer temperatures, increased rainfall, and humidity associated with ENSO events were found to enhance vector activity and disease transmission. Ocular complications included uveitis, retinopathy, and optic neuropathy, but the specific disease findings varied by infectious disease syndrome. The climactic variable changes in response to ENSO events differed across diseases and regions and were influenced by geography, local infrastructure, and socioeconomic factors. ENSO event-related climate shifts significantly impact the spread of infectious diseases with ocular symptoms. These findings highlight the need for region-specific surveillance and predictive models that may provide insight related to the risk of ophthalmic disease during ENSO events. Further research is needed to clarify long-term ENSO effects and develop integrated strategies for systemic and eye disease detection, prevention, and management.

## 1. Introduction

Since the 2015 Paris agreement, climate change has been increasingly reported to influence global health and has been integrated into the United Nations Sustainable Development Goals (UN SDGs) [1]. This integration into the UN SDGs is particularly relevant, given that the Earth’s temperature has risen by an average of 0.14° Fahrenheit (0.08° Celsius) per decade since 1880 [2], although climactic variables that extend beyond warming patterns are highly relevant to infectious disease syndromes. Recent studies have assessed whether fluctuations in precipitation, along with temperature patterns, may enhance the transmission of infectious diseases, including Dengue, malaria, and leishmaniasis, as well as several other infectious diseases [3]. Extreme weather events leading to extreme precipitation, infrastructure damage, and reduction in vector control, may have particularly profound impacts to vector-borne disease [4].

The ENSO climate phenomena, or ENSO events, involve the warming and cooling of ocean temperatures in the central and eastern equatorial Pacific, occurring alongside changes in the atmosphere that occur in cycles of approximately 2 to 7 years. This climate phenomenon consists of 3 phases: El Niño, La Niña, and a neutral phase [5]. An El Niño event is defined as a 12 to 18-month period of sea-surface warming, while a La Niña event is characterized by a period of colder-than-average sea surface temperatures in the central and eastern Pacific Ocean and an intensification of surface winds [5].

The extreme temperatures and climate patterns caused by ENSO have the potential to increase the number of outbreaks and cases of vector-borne illnesses. ENSO events often cycle between periods with abnormal increases in temperature and rainfall, particularly in Africa, Latin America, and South and Southeast Asia. During these ENSO events, the warmer and wetter conditions can impact vector behavior, including lengthened periods of travel for vectors and an increased likelihood of prolonged transmission of vector-borne illnesses [6]. Prior research has described the ophthalmic manifestations associated with multiple vector-borne illnesses, but the connection between El Niño events and eye disease has been rarely assessed in the ophthalmic literature [7].

We aimed to assess potential relationships between ENSO, climate events, and infectious disease outbreaks with ophthalmic consequences to better characterize the relationships between the ENSO phenomena and ocular health. As such, we conducted a systematic review of literature documenting notable infectious disease incidence and their correlation with ENSO events. We also assessed the ophthalmic manifestations of ENSO event-amplified diseases. Gaining a better understanding of the relationship between ENSO and ocular disease prevalence will inform our need as a discipline to proactively initiate appropriate surveillance measures for infectious diseases that may lead to ophthalmic findings and present risk to vision health.

## 2. Materials and Methods

We developed a narrative review based on a PubMed electronic database search to identify major outbreaks of each disease of interest and the potential associations with ENSO events. Our literature screening process was facilitated by Covidence software (Melbourne, VIC, Australia) and included articles published in English as of 11 July 2025. The PubMed search was cross-referenced with EMBASE, LILACS and Web of Science to identify additional relevant articles of interest. The literature search was updated on 25 September 2025. We then reviewed the relationship of these disease outbreaks to case series of infectious eye disease. Using infectious disease and ENSO-related terms, we identified 255 articles characterizing their association and potential association with infectious diseases. Terms utilized in our search related to ENSO included “El Niño”, “La Niña”, “ENSO”, and “El Niño Southern Oscillation”.

Search terms related to infectious diseases of interest for this report included “Dengue”, “Chikungunya”, “Zika”, “Rift Valley Fever”, “Malaria”, “Typhoid”, “Plague”, “Leishmaniasis”, and “Leptospirosis”. Two investigators (CMY and CH) conducted the initial prescreening, full-text screening, and data extraction process.

Thirty-five articles were selected for full review. Criteria for disease selection were determined based on the relationship between climate and disease transmission, global prevalence, and whether the infectious disease was associated with ocular manifestations. Articles selected for full review mentioned the disease of interest and its association with ENSO. Articles that did not specifically evaluate the relationship between ENSO and disease state were excluded. While the initial literature search initially included typhoid and plague, these pathogens are not summarized in detail owing to the limited data available related to the relationships between climate and ENSO with the ocular complications associated with typhoid and plague.

## 3. Results

**Figure 1:**
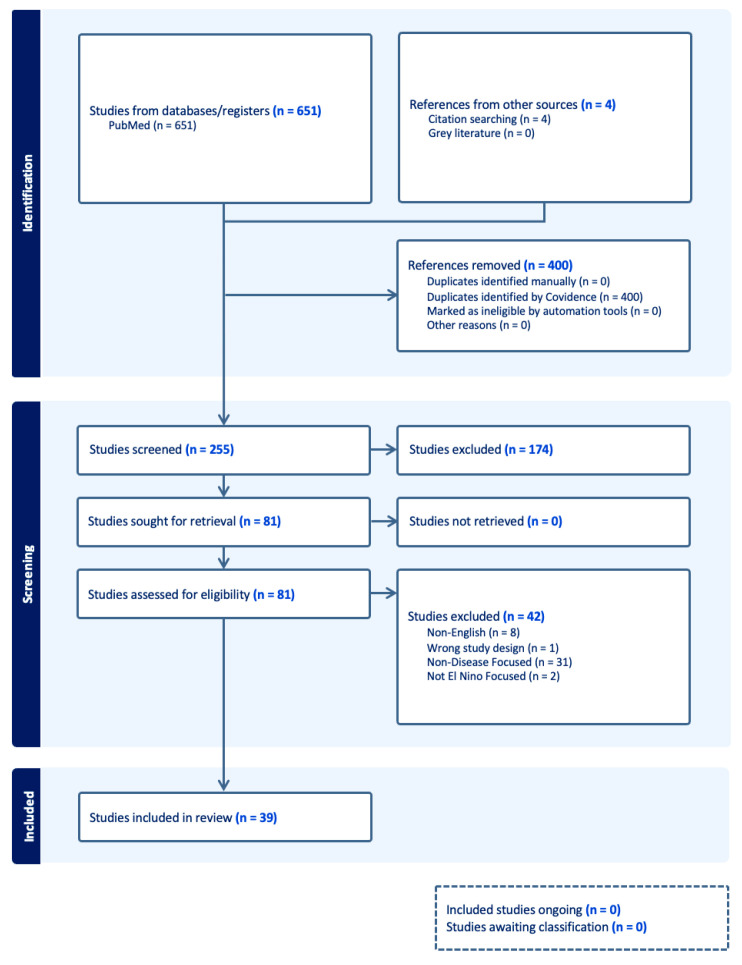
PRISMA Diagram.

Of the 255 articles identified, 39 articles were selected for full review (Figure 1, PRISMA Diagram). Most articles focused on Dengue virus (DENV) (14), while others focused on Malaria (10), Leptospirosis (5), Leishmaniasis (5), Zika Virus (ZIKV) (2), and Chikungunya CHIKV) (2). One article assessed the impact of ENSO or climate change on Rift Valley Fever (RVF). Given the relationships identified and numerous parallels between CHIKV, DENV, and ZIKV, we also summarized general considerations regarding the disease entities, climate change, and the potential impact of ENSO phenomena owing to their shared vector (i.e., *Aedes aegypti*), and the potential impact of ENSO and climate change on this vector-borne illnes (Supplemental Table S1).

### 3.1. Disease Transmission Considerations Related to Vector-Borne Illnesses: Mosquito and Tick-Borne Infectious Diseases

The primary transmission method of CHIKV, DENV, and ZIKV is via the *Aedes aegypti* mosquito, as humans and livestock can become infected following a mosquito bite. Outbreaks can be further amplified, as mosquitoes that bite infected humans are biological vectors capable of continued disease transmission [8].

The relationship between maternal ZIKV and newborn abnormalities is well-reported, but DENV and CHIKV also may manifest symptoms in infected mothers and their infants through neonatal infections [9]. Malaria, another mosquito-borne illness, is spread by the members of the *Anopheles* mosquito vectors, while West Nile Virus are transmitted by members of the *Culex* mosquito vectors. Rift Valley fever virus may be transmitted by multiple mosquito genera including *Culex*, *Aedes*, and *Anopheles* mosquito vectors [10].

**Figure 2:**
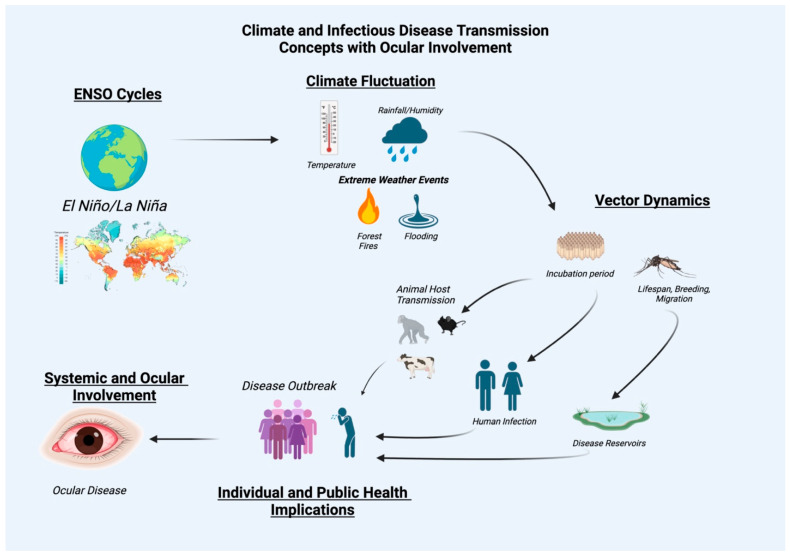
Relationships between El Nino Southern Oscillation events, climate and infectious disease transmission with ocular involvement as an infectious disease sequelae.

Ticks, fleas, and rodents are also vectors that may contribute largely to the distribution of diseases, including plague, rickettsial disease, leishmaniasis, and onchocerciasis. Prior studies have reported the relationship between climate and the behavior of these vectors [11]. Factors influencing vector-borne illnesses encompass a range of variables, including changes in temperature, precipitation patterns, and the availability of hosts [12,13]. These factors can greatly affect the life cycles of vectors, potentially extending or shortening their periods of activity and travel. Thus, ENSO events may heighten normal vector patterns of transmission, leading to shifts in disease prevalence and geographic distribution (Figure 2).

### 3.2. Dengue

#### 3.2.1. Epidemiology, Systemic and Features

**Figure 3:**
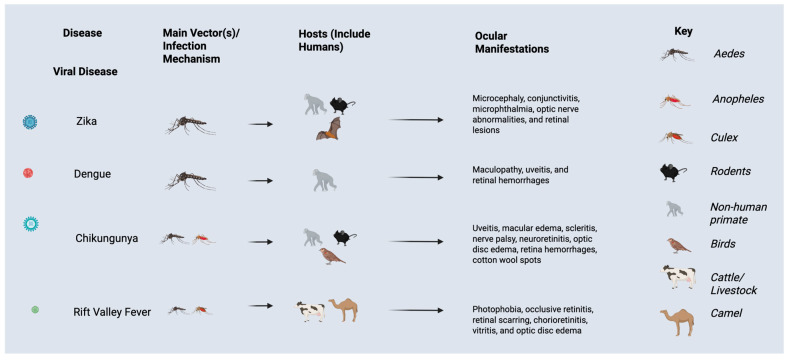
Ocular manifestations, vectors, and hosts of viral infections impacted by El Nino Southern Oscillation events and climate change.

Dengue manifests with sudden high fever, severe headache, retro-orbital pain, joint and muscle pain, rash, and bleeding. Prior guidelines from the World Health Organization (WHO) classified DENV infections into three stages: dengue fever (DF), dengue hemorrhagic fever (DHF), and dengue shock syndrome (DSS) [14]. The development of severe cases into DHF or DSS can be fatal. A range of ocular manifestations may be observed in patients with DENV infection (Figure 3), including dengue-related maculopathy, uveitis, and retinal hemorrhages, which can lead to vision impairment [15]. Subconjunctival hemorrhage and conjunctival petechial lesions may be observed, but vision may be unaffected in these cases [16,17]. Other symptoms may include retrobulbar pain and blurred vision [16].

#### 3.2.2. Impact of Climate: Vector Transmission, Temperature, Rainfall, and Humidity

The global risk of DENV outbreaks has risen steadily over the past four decades with the highest reproduction numbers (R0) in South America, southeast Asia, and equatorial Africa. ENSO was positively correlated with dengue risk in one study, although its impact varied by region and between events [18]. Transmission of DENV is highly influenced by climate, with warmer temperatures, increased rainfall, and increased humidity reported to promote mosquito breeding and lifespan, thereby enhancing virus spread [19]. Rising sea temperatures during ENSO exacerbated DENV cases in cities located along the coast of the Gulf of Mexico as well as in Suriname, Colombia, and the French Guiana region [20]. While climactic factors can contribute to the transmission of disease, potential non-climactic factors such as varying disease serotypes and herd immunity can impact vector populations and human exposure, particularly in areas of poverty or land use change [21]. Importantly, the relationships between climactic factors and mosquito abundance are non-linear, as extreme temperatures (i.e., either colder or warmer) can reduce mosquito populations. Additionally, extreme rainfall may initially flush breeding sites with a delayed effect of precipitation on mosquito populations as accumulation of standing water provides stable breeding locations. Moreover, the *Aedes aegypti* mosquito also prefers to lay its eggs in containers or household breeding sites, which can impact humans in close proximity [22].

Temperature fluctuations may also impact the entomological parameters related to the mosquitoes’ life cycle and the incubation period of DENV in female mosquitoes, while precipitation levels determine the availability of breeding sites [23]. Rising temperatures caused by ENSO events further lower the threshold for mosquito development, allowing for mosquitoes to breed at higher altitudes [24]. ENSO events have exacerbated DENV outbreaks with the associated increases of both temperatures and precipitation.

#### 3.2.3. Correlation of Outbreaks to El Niño/La Niña Events

Of the thirteen studies selected for full-text review, ten studies reported a correlation between a climatic factor and DENV cases. Climatic factors consisted of temperature, rainfall, humidity, and ENSO events. Multiple studies reported a lag of one to six months in the assessment of DENV with ENSO. This lag was deemed likely from a delay in transmission time as factors including external incubation (i.e., within mosquito) and internal incubation (i.e., within host) of DENV, may both occur to contribute to the rise in DENV cases [22]. Prior observations of a delayed contribution of climate to DENV cases are an important factor when forecasting DENV outbreaks.

Ferreria et al. observed a total of seven epidemic DENV outbreaks between 2007–2015 in Venezuela, four of which corresponded to El Niño events, while Vincenti-Gonzalez et al. reported results indicating that El Niño activity had occurred in four out of the five years in which an above-average number of Dengue cases was registered (1997, 1998, 2002 and 2003) [20,25]. Interestingly, Pramanik et al. reported that while El Niño influenced drier conditions in India, the increase in storage of water during these dry periods increased the sustainability of breeding sites for vectors [26]. Three studies reported a positive correlation between La Niña and incident DENV cases, including outbreaks in Venezuela, India, and the Solomon Islands. These areas experienced an exacerbation of monsoon and post-monsoon rainfall and an increase in temperature.

### 3.3. Zika

#### 3.3.1. Epidemiology, Systemic and Features

ZIKV infection may present with mild symptoms such as fever, rash, conjunctivitis, muscle and joint pain lasting for about a week. Severe complications can include Guillain–Barré syndrome. Congenital Zika syndrome (CZS) leads to serious birth defects and systemic symptoms in infants born to Zika-infected mothers [27].

In infants with CZS, ocular symptoms including microphthalmia, optic nerve abnormalities, and retinal damage, which can result in significant vision impairment or blindness, have been observed [28]. Conjunctivitis, chorioretinal scarring, and localized pigmentary changes in the macula have also been reported [28].

#### 3.3.2. Impact of Climate: Vector Transmission, Temperature, Rainfall, and Humidity

The transmission of ZIKV is influenced by climate conditions, with warmer temperatures and increased rainfall boosting mosquito populations. Similar to DENV, rainfall and temperature contribute to enhancing vector life spans by promoting breeding sites [29]. During periods of drought, *Aedes aegypti* mosquitoes lay eggs in domestic, water-storing containers, while in times of heavy rainfall, mosquito populations have copious environmental breeding sites [29].

#### 3.3.3. Correlation of Zika Virus Outbreaks to El Niño/La Niña Events

Caminade et al. (2017) observed major outbreaks on Yap Island in 2007, French Polynesia in 2013–2014, and Brazil in 2015–2016 [30]. A particularly notable ZIKV outbreak occurred in association with a 2015 El Niño event and was especially prominent with ZIKV transmission within South America [30]. The increase in transmission was associated with favorable climate conditions, such as an increase in rainfall and temperature, that dramatically increased biting rates and decreased mosquito mortality rates in 2015 [30]. These outbreaks were notable, as ZIKV infection was associated with numerous birth defects in infants, including CZS [28]. In addition, this outbreak was linked to increased cases of Guillain–Barré syndrome in adults. Muñoz et al. 2016 highlighted that while some extreme climate changes are not directly attributable to ENSO events alone, they can result from a combination of climate change and ENSO phenomena occurring on independent timelines [29]. Extreme climate anomalies associated with the ENSO from 2015–2016 created ecological conditions that favored outbreaks including Zika, dengue, and plague. Analysis showed an increase in disease activity varying from 2.5–28% in ENSO-affected regions compared to non-ENSO years [31]. Together, these studies underscore the interplay between climatic factors and ZIKV transmission, illustrating the multifaceted nature of outbreak dynamics.

### 3.4. Chikungunya

#### 3.4.1. Epidemiology, Systemic and Features

CHIKV consists of three stages: (1) Acute, (2) Post-acute, and (3) Chronic stage. During the acute stage, common symptoms consist of fever, rash, and bleeding gums. Optic neuritis has also been reported. The post-acute stage symptoms include inflammatory arthralgia, arthritis, and neuropathic pain. Finally, symptoms of the chronic stage include tendinitis, arthritis, and postural hypotension [32]. Ocular symptoms may include ocular pain and reduced visual acuity [33]. Anterior uveitis is the most common ophthalmic finding, while conditions like optic neuropathy, posterior synechiae, posterior uveitis, and macular edema have been described [8]. Additional ophthalmic features include keratitis, nystagmus, choroiditis, neuroretinitis, optic disc neuritis, vitritis, hyperemic disc, retinal hemorrhages, cotton wool spots, and multifocal retinitis [34].

#### 3.4.2. Impact of Climate: Vector Transmission, Temperature, Rainfall, and Humidity

No specific studies were identified in our review that found a correlation between ENSO and CHIKV transmission in endemic areas. While one study within Indonesia showed no significant effect of ENSO and CHIKV incidence, the Dipole Mode Index (DMI) showed a strong negative correlation with lower DMI values linked to higher cases [35].

#### 3.4.3. Correlating Outbreaks to El Niño/La Niña Events

CHIKV is of increasing global prevalence and has had a growing impact in the Americas since the first detected case in 2013 [36]. From 2021 to 2022, the reported prevalence of CHIKV rose from 1.2 to 2.8 million cases in the Americas [37]. Huang et al. 2019 analyzed the trends of CHIKV in combination with the ENSO and Southern Oscillation Index (SOI) during the time period from 2008 to 2017 [38]. The behavior of the virus reflects its patterns on a global scale as the disease has expanded both in prevalence and area affected during the time period. However, the article found inconsistent behavior of CHIKV prevalence relating to ENSO, suggesting that non-ENSO factors may play a larger role in disease burden within endemic countries. Within non-endemic regions, the study implied that the highest occurrence of vector-borne illnesses may occur during travel seasons. Specifically, when regions such as the United States, Papua New Guinea, and Bangladesh experienced outbreaks of CHIKV, Australia also experienced the highest number of reported cases.

### 3.5. Rift Valley Fever

#### 3.5.1. Epidemiology, Systemic and Features

RVF primarily affects livestock and humans on the African continent. RVF is transmitted through contact with infected bodily fluids or via the bite of an infected mosquito [39]. Around 10% of all patients develop symptoms including encephalitis, bleeding, muscle/joint pain, headache, and ocular manifestations such as eye pain, photophobia, and blurred vision [40]. Such symptoms may progress to severe visual impairment or blindness due to retinal vasculitis, macular or paramacular retinitis, retinal scarring, multifocal chorioretinitis, vitritis, retinal hemorrhage, and optic disc edema [7]. Non-granulomatous anterior uveitis may also be observed [41].

#### 3.5.2. Impact of Climate: Vector Transmission, Temperature, Rainfall, and Humidity

Because RVF’s common method of transmission occurs via mosquito vectors that include *Aedes* and *Culex* genera, El Niño/La Niña’s impact on vector habitats through climatic cycles may allow for a tangible relationship to be drawn between the ENSO phenomenon and the ocular symptoms. Primary hosts that are affected by vectors include cattle, goats, sheep, and camels, located primarily in eastern and southern Africa and the Arabian Peninsula [39].

#### 3.5.3. Correlating Outbreaks to El Niño/La Niña Events

RVF epidemics have also been closely tied to periods of heavy rainfall, so studies such as Oyas et al. have sought to examine a potential relationship between the warm phase of the ENSO phenomenon and RVF outbreaks [39]. This analysis was performed to assist in the development of predictive models to prepare different regions of Kenya in response to the expected burden of RVF. Using a Pearson’s correlation coefficient model, they were able to determine a positive correlation between RVF and rainfall/flooding, but interestingly, their study did not identify any documented RVF outbreaks during the surveillance period. This was deemed secondary to an absence of the expected ENSO rains during the epoch of interest.

### 3.6. Leptospirosis

#### 3.6.1. Epidemiology, Systemic and Features

**Figure 4:**
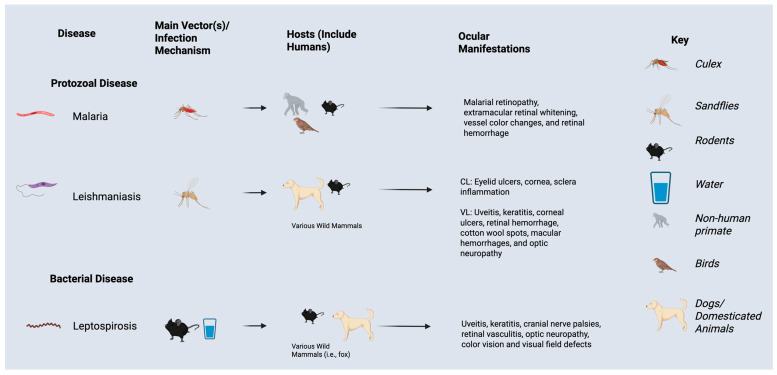
Ocular manifestations, vectors, and hosts of protozoal and bacterial infections impacted by El Nino Southern Oscillation events and climate change.

Leptospirosis, most concentrated in tropical/subtropical regions, affects a range of mammals, including cattle, dogs, cats, and marine life. Humans may contract these diseases through contact with urine or blood from infected individuals, or via contaminated soil and water [42]. Heavy flooding and rainfall can exacerbate disease prevalence via increased contact with Leptospira bacteria (Figure 4) [43].

Systemic symptoms include respiratory difficulties, fever, vomiting, diarrhea, jaundice, and rash. Kidney and liver damage or failure may also develop. Ophthalmic manifestations, including subconjunctival hemorrhage, scleral icterus, and conjunctival chemosis, may be observed. Other ophthalmic findings associated with leptospirosis include uveitis, keratitis, cranial nerve palsies, retinal vasculitis, and optic neuropathy with resultant visual field abnormalities or color vision deficits [44].

#### 3.6.2. Impact of Climate: Vector Transmission, Temperature, Rainfall, and Humidity

Weinberger et al. (2014) [43] reported that within the South Pacific Territory of New Caledonia, leptospirosis was found to be related more closely to Surface Sea Temperature Anomalies (SST) than ENSO events. Moreover, they proposed that accumulated rainfall may be the key driving factor between the leptospirosis vector and the increase in disease prevalence. Gutiérrez et al. (2018) found that cultural and socio-economic variables or even environmental factors besides rainfall, such as soil moisture, may play a larger role in leptospirosis burden [45]. The specific risk due to El Niño or La Niña may ultimately depend on the exact climatic effects on the region experienced during those events, potential exacerbation of vector species, and the major topography of the region of interest (i.e., river or impoverished town subject to flooding).

#### 3.6.3. Correlating Outbreaks to El Niño/La Niña Events

Four studies were selected through our literature search for leptospirosis for full review. Cross-correlation analysis was a method utilized by multiple authors to determine the association between ENSO and leptospirosis, which identified a relationship between La Niña events and leptospirosis burden [42,46,47]. Arias-Monslave et al. (2019) determined that there was a 25% rise in the monthly number of cases during La Niña periods, and a 17% decrease during El Niño periods [46]. The highest monthly number of cases was observed between 2010 and 2011, which was associated with a strong La Niña event. ENSO’s effect on flooding along the Cauca River, along with an increase in rodent vectors, illustrates a probable relationship leading to this correlation between ENSO and leptospirosis and its potential ocular manifestations. Ehelepola et al. further supported a potential relationship between La Niña and leptospirosis [42]. The study utilized wavelet analysis and cross-correlation, observing Sri Lanka’s Hambantota District from 2009 to 2017. Ehelepola et al. (2021) observed that seventeen cities experienced an increase of leptospirosis cases during La Nina events compared to only seven cities that experienced an increase of leptospirosis during El Nino events [42].

One other investigation of leptospirosis in Fiji from 2006–2017 found that cases increased within weeks after specific climate conditions. While heavy rainfall led to increased cases about six weeks later, cooler-than-average conditions influenced cases about four weeks later [48]. Moreover, the Western and Northern divisions of Fiji were more susceptible to climactic drivers, highlighting the potential for regional differences that could be relevant for public health measures [48].

### 3.7. Malaria

#### 3.7.1. Epidemiology, Systemic and Features

Malaria is a mosquito-borne, systemic disease caused by *Plasmodium* parasites and is heavily concentrated in the WHO African Region, where it accounts for the majority of global morbidity and mortality [49]. Clinical presentation ranges from uncomplicated, flu-like illness to life-threatening complications—severe anemia, acute kidney injury, cerebral involvement, seizures, and coma. Although ocular manifestations of malaria are uncommonly documented, previously defined ocular manifestations have included malarial retinopathy, extramacular retinal whitening, vessel color changes, and retinal hemorrhage [50]. These retinal changes provide valuable bedside clues to cerebral malaria and correlate with disease severity. The distribution and implementation of malaria preventative immunizations and response programs have been slowed by the COVID-19 pandemic, which, along with climatic cycles, has dramatically affected the global burden and distribution of malaria. Preventive strategies such as insecticide-treated bed nets (ITNs), indoor residual spraying (IRS), and seasonal malaria chemoprevention (SMC) were disrupted due to limited community outreach and healthcare access during the pandemic [49].

#### 3.7.2. Impact of Climate: Vector Transmission, Temperature, Rainfall, and Humidity

Malaria’s primary vector of transmission includes female mosquitoes of the *Anopheles* genus, which have activity patterns that are influenced by ecological and human-related activities. Delgato-Petrocelli et al. (2012) suggested that man-made alterations to the topography in Venezuela have enhanced the movement of vectors between areas and facilitated connections between large mosquito habitats [51]. Fluctuations in metabolic rate correlating to temperature may also contribute to changes in the production of vector offspring [51]. Additionally, the parasites responsible for malaria, *Plasmodium falciparum* and *Plasmodium vivax*, are highly influenced by climatic factors such as temperature, humidity, and rainfall, as well as seasonal changes [52,53].

#### 3.7.3. Correlating Outbreaks to El Niño/La Niña Events

Prior studies have evaluated the relationship between ENSO and malaria and found that their relationship was influenced by both the outbreak and the associated climatic variable. Delgado-Petrocelli et al. found a weak relationship between La Niña and an increase in Malaria cases in Venezuela from 1990 to 2000, while studies by Dhiman et al. (2017) and Hanf et al. (2011) in India and the Caribbean, respectively, found moderate relationships between the two variables [52,54].

The relationship between malaria and ENSO demonstrates that trends associated with ENSO events may be interpreted differently depending on the region. Variables such as inter-regional socio-economic trends and geospatial climatic behavior are other factors to consider in addressing the burden of malaria that may be altered by climatic events.

### 3.8. Leishmaniasis

#### 3.8.1. Epidemiology, Systemic and Features

Leishmaniasis is a parasitic disease caused by *Leishmania* parasites, transmitted through the bite of infected female sandflies [55]. Three forms of Leishmaniasis have been identified: Cutaneous leishmaniasis (CL), Mucocutaneous Leishmaniasis (ML), and visceral leishmaniasis (VL) [55]. The most apparent symptoms of CL are large ulcers, while ML most commonly damages mucous membranes of the nose, mouth, and throat. VL presents the highest morality rate as well as more severe systemic and ocular symptoms. Both CL and VL have been found to present differing ocular manifestations; CL most commonly impacts the anterior segment of the eye. The eye pathogenesis of CL results from ulcers of the eyelids, which can result in corneal and scleral inflammation, as well as keratitis. VL can affect more anatomic locations in the eye, including both anterior and posterior chambers. Ocular symptoms of VL include leishmanial uveitis, keratitis, corneal ulcers, retinal hemorrhage, cotton wool spots, macular hemorrhages, vascular sheathing, and optic neuropathy [56].

#### 3.8.2. Impact of Climate: Vector Transmission, Temperature, Rainfall, and Humidity

Leishmaniasis is commonly spread by the bite of an infected female sandflies that favor dry and warm climates. These conditions have been found to increase CL cases, while cold and wet conditions reduce vector population and density, decreasing the incidence of CL cases [57].

#### 3.8.3. Correlating Outbreaks to El Niño/La Niña Events

We identified two ENSO-related articles focusing on CL distribution and two articles dedicated to VL distribution. Articles focusing on simulation-based models or on the distribution of VL/CL vectors were not included in this review. All four leishmaniasis articles’ areas of study focused on cities in Brazil. Da Silva Neto et al. (2020) focused on VL and determined that there was no significant correlation, but that higher VL incidence was associated with La Niña events [58]. A decrease in VL cases during El Niño periods was attributed to El Niño’s effect on Mato Grosso do Sul, where increased rainfall reduced potential sandfly breeding grounds. However, Gutiérrez et al. (2024) observed in Brazil that both the 2010–2011 La Niña and the 2015–2016 El Niño led to increases in VL cases compared to neutral phases, with El Niño causing a substantially greater rise [59]. This discrepancy may be due to differences in study scale, as Da Silva Neto et al. (2020) focused on a single region, whereas Gutiérrez et al. examined both Brazil and Columbia.

Both studies on the relationship between CL and ENSO utilized similar methodologies, using wavelength coherence and phase difference analysis. However, Ferreira de Souza et al. (2015) determined that El Niño preceded higher cases by 6 months, while da Silva et al. (2021) found that end of the year La Niña occurrences would provide optimal conditions for CL increases during the start of the following year [57,60].

## 4. Discussion

This narrative review of the literature regarding the ENSO and infectious disease with ocular sequelae highlights the important role that rainfall, humidity, and temperature play in driving major disease outbreaks, particularly diseases with vector life cycles impacted by climactic variables. Increased rainfall was shown to provide sustained habitats for mosquito breeding, enhancing the longevity of seasonal cycles during which outbreaks of vector-borne diseases are most likely to occur.

Key relationships between humidity, temperature, and disease burden may be explained by how specific climactic variables may influence mosquito vectors associated with the infectious diseases of interest. Humidity plays a key role in extending mosquito lifespans by reducing the water lost through evaporation [61], while rising temperatures accelerate mosquito larvae development and increase overall mosquito activity. Different mosquito vectors, however, respond uniquely to environmental changes triggered by ENSO. By contrast, increased humidity has been identified as a key risk factor for the *Aedes aegypti* mosquito, noted as either a primary or secondary vector for the pathogens described in this review including DENV, ZIKV, CHIKV, RVF, and *Plasmodium species*.

The range of relationships observed in this literature review can likely be attributed to the variable effect of ENSO event periods, El Niño and La Niña, and the specificity of each respective cycle’s wide range of outcomes from climatic effects on regions. The specific impact of ENSO cycles makes it difficult to generalize the effect of the climate phenomenon’s relationship with global disease burden, emphasizing the importance of understanding region-specific responses. In India, to better predict malaria outbreaks, the effect of ENSO on the Indian Summer Monsoon Rainfall (ISMR) across the country was assessed. This study showed regional variability in the correlation between ENSO with ISMR. These correlation indices showed a positive correlation with a malaria case index in the eastern part of India, with negative correlations in the western part of India [54]. In Brazil, a study by de Oliveira-Júnior et al. (2019) found that the climate’s effect on increased DENV cases was also related to regional health policy and population growth [62].

Beyond climactic variables impacted by ENSO, other regional variables include population density, urbanization, and flooding in low-lying settlement areas associated with topography (e.g., coastal areas) may interact with the variations in climate associated with ENSO [63,64]. While ENSO events can lead to dramatic shifts in humidity, temperature, and precipitation, population density may increase transmission and hosts for disease, while water accumulation and flooding may increase mosquito breeding sites that are not driven entirely by the level of precipitation [65,66].

While the role of climate and extreme weather events has been evaluated for a range of infectious disease, the precise role of climactic factors on pathobiology on the eye itself has not been studied extensively. A relationship of decreased relative humidity and increased air pollution to dry eye disease has been observed [67,68]. Dry eye disease impacts the vulnerability of the eye to microbial keratitis [69]. Chaudhary et al. reported seasons of conjunctivitis that varied with local climate and air quality in Nepal with a peak of bacteria during pre-monsoon season, RNA viruses during monsoon season, and fungi during post monsoon season [70]. Studies to understand these relationships between environmental factors and conjunctival pathogens are needed.

Limitations encountered during our literature review included the lack of comparability between studies and the limited amount of research exploring the direct connection between ENSO and eye disease. Studies differed in their methodological approaches, including ENSO-related climatic variables assessed (i.e., temperature, humidity, air quality, or combination) and health outcomes examined. The geographic regions or populations studied also varied, making the studies difficult to generalize. In addition, the population density of the geographic regions was not universally assessed in the publications reviewed. Lastly, the majority of research focused on short-term effects, and longer term studies would be required to understand the extended impact of ENSO on both systemic and ophthalmic findings.

Despite these limitations, our findings revealed notable differences in how patterns of infectious diseases may vary in respond to El Niño and La Niña. Additional external factors, including travel rates and population density, may also play a critical role in transmission rates and remain key considerations when evaluating patterns of disease influenced by climate [38]. Given that ENSO events may ultimately impact the spread of the infectious disease where ocular disease may develop, either primarily or as a delayed sequelae, these findings highlight the importance of research that relates climatic risk factors associated with ocular disease in a range of infectious diseases, particularly vector-borne illness.

## 5. Conclusions

In conclusion, our review of the literature showed a range of climatic variables impacted by ENSO events—precipitation, humidity, and temperature—which may play a role in infectious disease outbreaks. The complex, region-specific impact on different vectors and pathogens, which can lead to both systemic morbidity and eye disease, highlights the need for detection, prevention, and management strategies that may vary by geography. While this current study has primarily focused on vector-borne diseases, our findings reveal significant gaps in the understanding of how ENSO affects ophthalmic infectious diseases more broadly.

Further research is needed to explore the biological mechanisms by which climate-related changes impact eye health, especially in regions more heavily affected by ENSO events. Improved predictive models that integrate both systemic and eye diseases will be crucial for better understanding and managing the public health impacts of these climate fluctuations. Addressing these research gaps will enable more effective prevention and surveillance strategies, ensuring that the full range of health outcomes influenced by ENSO are considered in future global health initiatives.

## Data Availability

No new data were created or analyzed in this study. Data sharing is not applicable to this article.

## Supplementary Materials

The following supporting information can be downloaded at: https://www.mdpi.com/article/10.3390/tropicalmed10100297/s1, Table S1. Summary of infectious disease syndromes, vector-pathogen relationships, clinical findings, and impact of climactic change and ENSO on disease transmission.
