# Supplementary material for: Impact of El Nino Southern Oscillation and Climate Change on Infectious Diseases with Ophthalmic Manifestations
Source: Trop Med Infect Dis. 2025 Oct 18;10(10):297. doi: 10.3390/tropicalmed10100297 (PMC12567761; doi:10.3390/tropicalmed10100297)
Supplement: Supplementary file 1 [file tropicalmed-10-00297-s001.zip › tropicalmed-3798170-supplementary.pdf]

Table S1. Summary of infectious disease syndromes, vector-pathogen relationships, clinical findings, and impact of climactic change and ENSO on disease transmission.

**Infectious Disease syndrome**	**Causative pathogen(s)**	**Vector**	**Systemic findings**	**Ophthalmic findings**	**Implications of climactic variables and ENSO events on disease transmission**
Dengue	Dengue virus (DENV)	*Aedes aegypti*	High fever, severe headache, muscle pain, rash, bleeding Dengue Fever, Dengue Hemorrhagic Fever, Dengue shock syndrome	Retro-orbital pain, maculopathy, uveitis, retinal hemorrhage, which can lead to vision impairment	Warmer temperatures, rainfall, and humidity increase mosquito breeding [18]<br><br>Warmer temperature can increase mosquito life cycle and incubation period of DENV in female mosquitoes [21]<br><br>Precipitation increases breeding sites of mosquitoes [21]
Chikungunya	Chikungunya virus (CHIKV)	*Aedes aegypti*	Acute: Fever, rash, bleeding gums<br>Post-acute: Inflammatory arthralgia, arthritis, and joint pain<br>Chronic: Tendinitis, arthritis and postural hypotension	Anterior uveitis, posterior synechiae, posterior uveitis, macular edema [7,8], keratitis, neuroretinitis, optic disc edema, retinal hemorrhages, multifocal retinitis	Inconsistent behavior of CHIKV prevalence relative to ENSO, suggesting that non-ENSO factors may play a larger role in disease burden
Zika	Zika virus (ZIKV)	*Aedes aegypti*	Fever, rash, joint and muscle pain ~ 1-week duration; Guillan-Barre syndrome may develop as a late finding	Conjunctivitis during acute disease<br><br>Congenital Zika virus syndrome: microphthalmia, optic nerve damage, chorioretinal scarring in infants born to ZIKV-infected mothers	Warmer temperature and precipitation increase mosquito populations for ZIKV transmission [30]<br><br>Increased breeding sites with increased temperature and precipitation [29]<br><br>ENSO event correlated with ZIKV outbreak in 2015 (e.g., increased biting rates and decreased mosquito mortality) [30]
Rift valley fever	Rift valley fever virus	*Aedes* and *Culex* mosquitoes	Headache, encephalitis, muscle aches/pains, bleeding	Retinal vasculitis, retinitis, multifocal chorioretinitis, vitritis, retinal hemorrhage and optic disc edema	Correlation between Rift valley fever and rainfall and flooding [39]
Leptospirosis	*Leptospira* bacteria	Acquired via contact with infected animals (e.g., dogs, cats, cattle), infected urine or blood, or via contaminated soil and water	Fever, vomiting, diarrhea, jaundice, rash, kidney and liver damage may develop	Subconjunctival hemorrhage, scleral icterus, uveitis, keratitis, cranial nerve palsies, retinal vasculitis, optic neuropathy	Increased number of cases during La Nina / Decreased cases during El Nino [46]<br><br>High number of cases has been associated with a La Nina event [46]<br><br>Increased leptospirosis in 17 cities during La Nina compared to 7 cities during an El Nino event [42]
Malaria	*Plasmodium falciparum, Plasmodium vivax*	Female mosquitoes of *Anopheles* genus	Clinical features range from uncomplicated flu-like illness to life-threatening complications (cerebral malaria,	Malarial retinopathy, extramacular whitening, retinal vascular color changes, retinal hemorrhage	Relationships observed between La Nina events and increased malaria cases in Venezuela [52]

			anemia, seizures, coma)		
Leishmaniasis	*Leishmania* parasites	Female sandflies	Three forms: Cutaneous leishmaniasis (CL); Visceral leishmaniasis (VL); Mucocutaneous leishmaniasis (ML)	CL: Ulcerations of eyelids, keratitis and scleritis<br><br>VL: Anterior and posterior segment eye disease – Uveitis, scleritis, keratitis, corneal ulcers, cotton wool spots, optic neuropathy	VL: Higher VL incidence associated with La Nina events, not a significant correlation<br><br>Decreased cases during El Nino period due to increased rainfall and reduction in sandfly breeding grounds<br><br>One study reported a greater number of VL associated with El Nino *and* La Nina, with greater rise associated wih El Nino

Abbreviations ENSO El Nino Southern Oscillation, DENV Dengue virus, ZIKV Zika virus, CHIKV Chikungunya virus, CL Cutaneous leishmaniasis, VL Visceral leishmaniasis, ML Mucocutaneous leishmaniasis
